# Closing the Gap: From Research to Practice in Mental Health Interventions

**DOI:** 10.3390/ijerph20032141

**Published:** 2023-01-24

**Authors:** Lena Lipskaya-Velikovsky, Terry Krupa

**Affiliations:** 1 School of Occupational Therapy, Faculty of Medicine, The Hebrew University, Jerusalem 9124001, Israel; 2 School of Rehabilitation Therapy, Queen’s University, Kingston, ON K7L 3N6, Canada

**Keywords:** participation in daily life, occupation-oriented intervention, effectiveness, clinical practice, inpatient setting, knowledge translation

## Abstract

Evidence-based practice is critical but challenging in mental health. Rigorous research-proven interventions often do not yield expected results in the clinical practice. This study aimed to explore factors contributing to the effectiveness of Occupational Connections (OC)—an intervention for promotion of engagement in meaningful occupations in serious mental illness (SMI)—based on case series study of three quasi-experimental studies. The studies focused on people with SMI, admitted to intensive mental health services participated in the OC, as well as on a control condition group. Similar evaluation procedures throughout these studies addressed primary outcomes of participation dimensions and recovery orientation, as well as secondary outcomes of functional capacity, cognition, and symptom severity. Patterns of changes in outcome measures varied between the three studies as to direction and extension. In the OC groups, 29–60% of the outcome measurements were changed, in comparison to 29–43% of measurements in the control groups. The secondary outcomes were consistently improved in the OC (18–100% of measurements) in comparison to the control (18–67%). The analysis of the studies revealed that clinical effectiveness of participation-oriented intervention varied dependent on interplay between the clinical context, clinician actions, served persons’ characteristics, and evidence-building process. These factors should be considered to maximize research benefits for practice.

## 1. Introduction

Recovery vision guides mental health services, which should be supportive of individuals in the multidimensional process of change toward living a self-directed life and reaching their full potential with mental health conditions [1,2]. Following that, one of the key tenets of professional practice is providing respective, flexible, and responsive services to client needs, values, and beliefs. In other words, a personally-centered and individually-tailored intervention is needed to support a personal journey of recovery [1,2]. At the same time, for a decade, there has been an expectation for the integration of research-based practices into this process in order to ensure high-quality, effective service [3,4]. However, there has been continuing debate regarding what this integration would mean and how it can be done, both in general and in the field of mental health services guided by recovery vision in particular.

Service providers/clinicians may feel conflicted working in accordance with basic philosophical principles of person-centered and individually tailored practices to meet a person’s unique needs and preferences while also trying to adhere to research-grounded intervention procedures in a specific practice context or service [5]. Practically, they may find themselves facing substantial questions, e.g., whether interventions should be applied rigorously, exactly as performed—frequently under laboratory conditions, in research—or a flexible implementation might still be acceptable. If so, to what extent or under what conditions is flexibility admissible? To what extent might the intervention undergo changes and still be considered the same (in order to be supported by evidence and be potentially reproducible) [5]? 

Rigorous implementation of research-grounded procedures was one of the Achilles’ heels of early conceptualization of evidence-based (EB) practice. Today’s vision of EB practice is an integration of research evidence, clinical expertise and experience, using client preferences to best tailor interventions to every person [3]. This vision supplies conceptual answers to questions while still providing little tools for practice. Furthermore, the literature has focused on describing productive processes for evidence establishment [5], referring less often to issues and tensions of future non-research-related implementations of interventions. There are few guidelines and tools to help clinicians skillfully utilize research-based evidence in practice [6]. In other words, the current definition of EB has left a gulf in translation of research into practical implementation—known as the research-to-practice gap—which is particularly prominent in mental health.

Issues of the research-to-practice gap are intensively discussed under the umbrella of the knowledge translation (KT) process, which is dedicated to maximizing research benefits for practice by providing relevant information for clinical decision-making [7,8,9]. The discussion in the field of KT may be divided into two components: (1) knowledge creation and (2) action-in-practice, based on the knowledge-to-action (KTA) process conceptual framework [8]. Each component includes several phases, with no definite boundaries between the two components and among their phases. The process of KT is complex and dynamic, depending on multiple factors which mutually influence one another [8,10]. In this way, the KT process involves, essentially, all stakeholders (researchers, policy makers, funds, clinicians, clients, and so on), requiring exchanges between those who create new knowledge and those who use it to improve health outcomes [9,10]. Even though the KT process has been discussed, there is little in the literature relating to the field of mental health. The purpose of this work was to examine the impact of various factors, across different phases of knowledge creation and action-in-practice processes, on clinical effectiveness, using examples of an intervention known as Occupational Connections (OC). OC [11,12] is intended to meet the occupation-related and recovery-promoting needs of people with mental health conditions in intensive care, with respect to their unique interests, values, needs, and experiences. This type of intervention is under-investigated in psychiatric in-patient settings. An important role of the psychiatric inpatient setting is support of reintegration into community life, including involvement in everyday occupations. The effectiveness of that role was estimated through changes in primary and secondary outcomes of the following aspects: participation, experience of the service as recovery-oriented, functional capacity, cognitive functioning, and symptom severity.

## 2. Materials and Methods

To meet our aim, we used three quasi-experimental, single-blind studies with convenience sampling on the effectiveness of the OC intervention developed for psychiatric inpatient settings as a case study. The first two studies were of the pre-post, comparative design, with study groups participating in the OC intervention in addition to receiving treatment as usual (TAU) in the hospital and control groups receiving TAU with the addition of one psychoeducational or informative group. The design of the third study was a crossover. The evaluations addressed similar constructs throughout the studies, with complete equivalence in the first and second ones. All three studies were designed based on the results of the pilot study [11]. To determine the impacts of different factors on KTA processes, we compared patterns of change in outcome measurements that were observed through the studies.

### 2.1. Participants

All three studies included participants with serious mental illness (for further details see Table 1), aged 18–60, who received intensive mental health services (in-patient stays or a five-day-a-week ambulatory program) and experienced subjective and/or objective restrictions in participation in a range of daily life activities. The exclusion criteria for the studies were co-occurring developmental and/or acquired neurological conditions, physical disabilities, and current substance/alcohol abuse.

#### 2.1.1. Study 1

This study involved mostly women in their 30s, all of whom had prolonged experience living with serious mental illness, mainly schizophrenia, and had been hospitalized in acute wards of a regional mental health center (N = 33) (Table 1). No differences were found between the study and the control groups in the demographic- and illness-related variables, except for the number of previous hospital admissions, which was significantly higher for the study group [12].

#### 2.1.2. Study 2

The study’s participants were mostly men. Like the first study’s participants, they were in their 30s, had prolonged experience of living with serious mental illness (mainly schizophrenia), and had been hospitalized in acute wards (N = 30) (Table 1). There were no statistically significant differences between the study and the control groups in the demographic and illness-related data [13].

#### 2.1.3. Study 3

In this study, more than half of the participants were women in their 30s. Differing from the previous studies, prevalent diagnoses in this study were affective and anxiety disorders. The participants were admitted to day-care programs throughout the study, and had less prolonged illness in comparison to participants of the other two studies (N = 19) (Table 1).

### 2.2. Context

The studies were approved by an Institutional Review Board of the Ministry of Health (BY-280; SHA-13-0009; RMB-14-0575, in accordance with the study’s number) and were conducted in accordance with the Declaration of Helsinki. All the studies’ participants provided written informed consent. Each study was conducted in different mental health regional centers, operated by 3 different health organizations, which varied as to regulations, policies, staff positions, etc., and covered different geographical and socioeconomical regions. The first study took place in the mental health center where the intervention was originally developed. The evidence for the intervention’s effectiveness in this context was previously reported [12]. The studies were performed with a single-blind procedure by licensed occupational therapists (OTs). Different OTs, through the sites, delivered the intervention.

### 2.3. Measurements

The intervention’s effectiveness was investigated in accordance with its theoretical background. The primary outcomes were set with a focus on participation and recovery dimensions that were addressed with (I) the Intention to Participate Scale for evaluation of the intention to participate in meaningful activities, (II) the Impact on Participation and Autonomy tool (IPA), assessing experience of autonomy in activities and occupations, and (III) the Recovery Self-Assessment for evaluation of the experience of service as recovery-oriented. The secondary outcomes addressed personal factors and included measurement of (I) dimensions of participation in daily occupations, as assessed with the Adults Subjective Assessment of Participation, (II) functional capacity in the IADL (assessed with the Observed Tasks of Daily Living-Revised, (III) aspects of cognitive functioning (evaluated using the Neurobehavioral State Cognitive Assessment; Trail Making Test, Part A and Part B; Rey-Osterrieth Complex Figure test; and Category Fluency Test) or general cognitive status (evaluated with Modified Mini-Mental State Examination), and (IV) the severity of the illness’ symptoms (the Positive and Negative Syndrome Scale), for first and second studies only. The detailed description of the evaluation procedures and tools can be found in our previous publications [11,12] and are attached in the Supplementary Materials, File S1. The summary of the studies’ procedures is presented in Table 1.

### 2.4. Intervention

Occupational Connections OC [11,12] was designed to promote engagement in occupations with personal and social meaning, to encourage understanding of occupational experiences, and to expand positive aspects of the experience of people with mental illness. As it was grounded on the Canadian Model of Occupational Performance and Engagement and the recovery model, one of the basic assumptions of OC is that participation in personally- and communally-meaningful occupations is an important dimension of the recovery journey [1]. OC was developed for an inpatient setting and further expanded for additional types of intensive psychiatric care. OC was implemented as a cyclic open group, with 10 sessions in each cycle, once or twice a week. Each session focused on a specific topic (such as experience of meaning in occupation, identification of possibilities for participation, finances, and participation) that was fully processed in the same session. The sessions lasted about 45 min and had a similar structure to support the group format. For example, every session began with individual narratives of occupational experience and ended with building an individual plan for work outside of the group sessions to enable attainment of personal aims related to meaningful occupations and participation patterns. The intervention was delivered based on a detailed manual, providing theoretical and practical materials for work and guidelines for enhancing the relevance of sessions for each participant. In addition, the intervention included an information kit for the multidisciplinary staff of inpatient settings to encourage their involvement in the promotion of positive occupational patterns [12]. In the third study, the protocol of the intervention was adapted to the structure of the service and the clients’ characteristics, through the standard procedure, using focus groups. In the process of adaptation, two sessions were omitted from the original manual. However, all participants completed all eight sessions.

The OTs who provided the intervention (different ones for each study) received supervision in different formats, based upon their availability (Table 1). The fidelity registration table was developed for the intervention and was completed after each session in all studies. We assumed that, within 5–8 weeks of the intervention, the results would be reflected in subjective dimensions of participation and experience of the service as recovery-oriented. Thus, these measures were set as the primary outcomes. We expected little change in the secondary outcomes of functional capacity, cognition, and symptoms, since no direct training was done on these skills. In addition, we did not assume extensive changes in the objective participation dimensions because of restrictive features of the in-patient environment and the intensive day-care program.

### 2.5. Analysis

The data were analyzed using SPSS 27. Descriptive statistics were used to characterize the participants. The control was done for demographic- and illness-related parameters that were found to be different at the baseline. The distribution of continuous data was estimated first using the Kolmogorov–Smirnov Test, and was reapproved for the purposes of this study with the Shapiro–Wilks test. The data from the first and second study were analyzed as follows: pre/post differences were analyzed using the Paired-samples *t*-test for data with normal distribution—otherwise, the Wilcoxon test was used. Between-group differences were analyzed using the Independent Samples *t*-test for data with normal distribution and with the Mann–Whitney test for data with other than normal distribution. The data from the third study were analyzed using ANOVA repeated measures. We used the Benjamini–Hochberg correction for assumptions, which included several comparisons. For the purpose of this work, we presented trends of change in the outcome measures based on statistical analysis, after controlling for the impact of the demographic- and illness-related data and the percentage of outcome measures that reached a statistically significant change (either improvement or decrease). The percent was calculated based on the following information. The primary outcomes addressed participation dimensions and recovery orientation of the service and included a total of 7 measures for studies 1 and 2, and 5 measures for study 3. The secondary outcomes addressed cognition (12 measures for studies 1 and 2, 1 measure for study 3), symptom severity (3 measures for studies 1 and 2), functional capacity (1 measure for all studies) and current participation patterns (4 measures for all studies).

## 3. Results

Different patterns of change in pre- and post-measurements were observed through the studies in the primary and secondary outcomes. These will be a subject for further discussion of KT processes.

### 3.1. Study 1

Following the OC intervention, improvement was found in primary outcomes of intention for participation in meaningful activities (IP) and experience of the inpatient service as recovery-oriented (RSA), but not in the experience of autonomy (IPA). Altogether, 29% of primary outcomes were improved in the study group, but no changes in primary outcomes were found in the control group. In addition, in this study group, improvements were observed for secondary outcomes of cognitive functioning (27% of the cognitive measurements), all types of symptoms (100%), functional capacity (100%), and participation diversity (25% of the participation dimensions’ scores). Little improvement was found in the secondary outcome measures in the control group (8% of cognitive measurements and 33% of the symptoms) [12] (Table 2).

### 3.2. Study 2

A decline was found in this study group in primary outcomes of intention to participate and experience of autonomy in family role-related activities (29% of primary outcomes measurement). However, in the control group, we found an increase in experience of the service as recovery oriented, and in experience of autonomy in indoor activities and social life-related activities (43% of the outcomes). As for the secondary outcomes, both groups improved on cognitive functions (18% of all cognitive measures for each group), even though the improved skills differed between groups. A similar extent of improvement in symptom severity was found in the two groups (67% of measures). However, only the study group improved in functional capacity (100%). No improvement in participation dimension was found in either group [13] (Table 2).

### 3.3. Study 3

Following the OC intervention, improvement was noted in primary outcomes of experience of autonomy in outdoor activities, work, and education activities and in social life-related activities (60% of outcome measures). No changes in primary outcomes were found in the control condition. Among secondary outcomes, participation in the intervention contributed to improvement of functional capacity (100%) and participation diversity and enjoyment (50% of participation dimensions’ scores), but not to general cognitive status. No improvements in secondary outcomes were found in the control condition (Table 2).

## 4. Discussion

Today, decades after establishing the EB practice concept, a knowledge-to-practice gap exists [8,10]. This situation is even more complicated in the field of mental health, since (1) recovery-supportive intervention should be personally centered and flexible [1,2] and (2) the evidence on outcomes of many interventions is not binary (i.e., whether the intervention is effective or ineffective). These factors, taken together, suggest that a more nuanced approach is needed for interpretation and skilled implementation of the knowledge [10]. This case series of three effectiveness studies of occupation- and recovery-oriented intervention for intensive mental health settings was used to elaborate on the impact of various factors throughout the process of research, evidence creation, and practical implementation procedures, and the extent to which the planned outcomes may be obtained in practice. The insights were discussed, focusing on different issues relating to knowledge creation and action.

### 4.1. Action for the Knowledge Implementation

The findings demonstrated that the OC intervention could be used in various settings with different groups of populations having target characteristics. However, the patterns of change in primary and secondary outcomes could be seen as different throughout the three studies. These findings further supported the notion of effectiveness of previously established interventions in a new context. Still, a thorough consideration of a range of intervention qualities and contextual factors (in terms of settings, clinician, and client) are needed to obtain the planned outcomes [6].

One factor that has a strong impact on multiple aspects of the intervention implementation and should be addressed is setting features [6,14]. Indeed, based on the descriptively-presented trends, it could be concluded that the three presented studies produced different outcomes, occurring in different settings, e.g., service structures, policies, OT staff actions, etc. We learned that the similarity of the settings, as judged by a basic definition such as “inpatient acute setting”, did not ensure similarity between the settings in characteristics deemed relevant for the intervention. These findings implied that complex interventions, such as the OC, which involve additional personnel and service infrastructure usage, are particularly sensitive to setting contingencies. Thus, a thorough consideration of the unique setting’s features should be done for intervention implementation in each new setting. On the other hand, the results suggested that, in the process of selecting new practices to be transported, no intervention should be ruled out based on a definition of the basic setting. In our work, we found that the OC, originally developed for inpatient settings, was able to yield the planned outcomes in day-care programs. To help clinicians ensure an intervention–setting fit, several structured models were suggested [6,9,14,15]. Even though many of them provide primarily general, conceptual tools, they may be useful for understanding the scope of the affecting factors and required resources for KTA activities. In the case of the OC, we found that critical factors for the intervention-setting fit included convergence between the OC aims and the service goals, general staff knowledge of activity benefits for health, actions taken by staff to support health through participation in meaningful activities, and an environment that enable practicing of personally relevant activities and goals. However, it is important to note that, in the process of intervention-implementation planning, settings’ features are in tight interplay with additional contextual factors [14,15].

The discrepancy in the studies’ results could be explained by the clinician/group facilitator (GF) position regarding the intervention–implementation process. Even though all the GFs had similar professional backgrounds throughout the studies and completed a workshop on OC implementation, there were differences. In the first study, the GF received direct modeling and ongoing supervision from one of the intervention authors in order to keep the intervention focused on key constructs. Meanwhile, in the second study, implementation was mainly based on the manual, with adherence to the rigorous research procedures, in order to keep similarities between the studies. Interestingly, prominent differences in the outcome measures were found between the first and second studies, supporting the notion that direct adherence to the technical aspects of the intervention, based primarily on the manual, could lead to a less flexible and manual-centered implementation, thereby limiting applications of clinical reasoning and yielding only partial outcomes [6,9,16]. Indeed, it was demonstrated that incorporation of a new intervention into practice was a complicated and dynamic process; valuable implementation may be best supported by an ongoing building of capacity for the practice, including intervention delivery issues and actions relating to intervention adaptation [6,15]. Capacity building, which is performed through a continuous process of thinking and reflection on the intervention, can enable the development of strong commitment to the intervention’s key constructs and concepts, rather than to the technical procedures only, and contribute to the achievement of a wide range of relevant primary and secondary outcomes [8,14,15,17,18,19]. In light of this capacity building concept and the spiral nature of the KT process in long-term implementation, this case study raised the question of what an appropriate stage might be to measure the intervention’s effectiveness through research on the continuum of the intervention delivery. The findings suggested that an investigation on the effectiveness of practice could be more trustworthy at advanced stages of implementation, contributing in a more valuable way to decisions regarding its clinical utility.

An additional approach for GF to serve as an implementation agent is to initiate the process of planned adaptation in the preparation phase [14,15], the action strategy applied in the third study. The adapted procedures yielded improvement in primary outcomes while still contributing to the improvement of secondary outcomes, demonstrating the effectiveness of this strategy to obtain planned outcomes. This served an additional example of the negotiation between direct adherence to research-approved procedures versus flexible implementation of the intervention while acknowledging the practitioners’ and clients’ values and goals and responding to the contextual aspects of practice. Still, effective and powerful tools are required in this situation to maintain balance between the extent of changes that may be accepted, within the boundaries of each intervention, butstill be supported by the evidence, with the potential to reproduce similar outcomes [5]. In addition, long-term, effective implementation may require further adaptations; thus, ongoing consideration of optimal implementation procedures is needed [15], suggesting the importance of a combination of strategies for beneficial integration of a new intervention in clinical practice [20].

One of the tools that could help clinicians keep within the intervention boundaries while adopting the intervention in a new context is the fidelity registration procedure. The concept of intervention fidelity refers to the degree with which the intervention is implemented as planned or intended, based on theoretical assumptions and practical approaches and aspects [21,22]. Originally articulated in the field of controlled studies, it has been less noticeable in clinical practice [23,24], raising further questions about its clinical applications. Indeed, even though a fidelity registration form was developed and kept for the OC intervention, it seemed to be insufficient, given the evident differences between the three studies in the outcome measures. These findings further illustrated the complexity of fidelity and the need for its broader vision in clinical practice, especially in cases involving complex interventions and outcomes which might require responsiveness to ever-changing life circumstances and narratively-described client experiences [25]. The case study alluded to the need for a shift in the process of fidelity form design, referring mostly to technical aspects of intervention structure and process. This over-emphasized their importance, putting the intervention integrity at risk and, likewise, its effectiveness toward a focus on conceptual intervention issues [26]. In fact, fidelity should (1) address issues, such as the main constructs and concepts underlying the intervention and guiding actions, and (2) specify mechanisms of change and ways to obtain it [24], formulating active components of the intervention. On behalf of the action side, clinicians should (1) identify/recognize active components of the intervention and (2) main features of research evidence, and (3) adapt the intervention in a skilled manner, based on obtained insights, context, served population, etc. [17]. Another issue may be covered by fidelity procedures, reflecting the complexity of intervention procedures toward planned outcomes. One example (in the case of an OC) may be the importance of the group climate and the shared emotional connection between the group participants, and the process of collaboratively working on a range of personal goals during a group session. Fidelity registration could help to ensure the integrity of the intervention and faithful-to-content implementation, with a higher probability of obtaining optimal outcomes [20,24]. In addition, usage of such fidelity procedures will contribute to the ongoing process of capacity building. The systematic documentation of the practice, with reflection on arising issues, will contribute to the researcher–clinician knowledge exchange and further the building of evidence in a supportive way for clinical practice.

### 4.2. Knowledge Creation

The results of the three studies demonstrated different patterns of change in primary and secondary outcome measurements, up to reversal, supporting the notion of contextual impact on intervention effectiveness [5,6,8,9,14,16]. These findings may be seen as challenging to the traditional vision of rigorous research procedures, with a strict adherence to standard intervention practices, in all contexts, in order to obtain further proof of the intervention effectiveness and demonstrate generalizability of the results [25]. In our view, these findings helped to extract supportive research procedures for clinical practice, demonstrating the importance of replicating effectiveness studies in a naturalistic manner, in different contexts and with different groups of target populations. This should take place as early as the initial stage of evidence building, in order to obtain information about practitioner and client and gain perspective on the intervention [5,9]. This information will be critical for (1) knowledge creation, as it may inform appropriate research designs and outcome measures for rigorous methodology studies [27,28]. The results of our previous studies serve as an example, demonstrating the limited relevance of the well-being measure as an immediate intervention outcome measure [11], and highlighting reasons to address decreases in subjective measurements as improvements, following the intervention (e.g., change in detached experience of autonomy in daily life activities) [13]. Moreover, replicating effectiveness studies could enhance (2) clinical implementation, delineating the understanding of which types of outcomes, with what population and under what conditions, could be obtained [5,6,14]. The OC effectiveness studies suggested that, at the sub-acute stage of mental illness, with low dosages of intervention, objective parameters such as functional capacity, cognitive functions, and symptom severity may be expected to improve, rather than subjective parameters such as the experience of autonomy in occupations and the intention to participate. On the other hand, with people at advanced stages of symptom stabilization, with actual involvement in everyday life and higher intervention dosages, such as those in day-care programs, improvements in subjective parameters related to occupation and participation, as well as functional capacity improvement, may be expected, rather than changes in cognitive functions. In addition, it was demonstrated that the pattern of improvement in outcomes following the OC intervention, with focus on reflection, participation aspects, and issues, rather than on doing or direct skills training, could depend on the participants’ gender. Throughout these studies, subjective outcomes were most improved among women, while more objective parameters were improved among men, suggesting a further need to investigate the relevance of different types of participation and occupational balance-related outcomes by gender. Cumulative findings from these studies provided further support for a complex interplay between the clients’ characteristics, intervention implementation, and expected outcomes that should be considered by clinicians in the process of skilled intervention implementation. To enable skilled implementation through adaptation while reducing the risk of deviation from the initial intervention principal components (thus, interrupting the fidelity), the knowledge creation process should involve the development of structured but flexible guidelines providing practical tools to address implementation challenges in various contexts [25]. Moreover, as was previously delineated, the supportive evidence-building process should be applied to reveal the impacts of various contextual factors, supporting further development of clinical guidelines as a powerful tool in helping to overcome the contextual barriers of implementation and closing the gap between the research and the clinic [5,9,19]. In the case of the OC intervention, the package to expand general staff knowledge on participation and activity benefits to health was developed to address contextual impacts on the intervention’s effectiveness. However, the findings suggested that this step was insufficient to support the OC implementation across a range of contexts. Next, following the previous discussion, we suggested including guidelines for clear statements on the active intervention components and developing fidelity methods beyond descriptions of the technical procedures dedicated for clinical practice [25]. The OC manual was designed as a mix of detailed descriptions of specific practices, but a range of materials was developed for each stage to meet participants’ personal needs and preferences. Moreover, for each intervention topic, an overarching goal was articulated to focus the practice. Still, further development of the manual will be needed to incorporate new understanding gained from this case study.

This serial case study had several limitations. First of all, the studies that served as the cases for the study were limited in sample size and their methodological issues were quasi-experimental; these factors all had the potential to alter the original study’s results. In addition, the contextual differences between the studies were not an issue of manipulation through the study design, but the results of a naturalistic approach, and thus limited a systematic comparison. Moreover, we only addressed one intervention through the series of cases. Different interventions with different scopes and aims, theoretical backgrounds, and practices could have yielded different results and been sensitive in different ways to the impact of the contextual factors on the process of knowledge creation and implementation. We investigated differences in the patterns of change in outcome measures only descriptively; this was a potential subject for bias and should be further assessed statistically.

## 5. Conclusions

To summarize, the adoption of new evidence-based interventions is imperative to expand the scope of practices to support health and well-being. This case series illustrated the complexity of knowledge creation and clinical action processes oriented toward the achievement of optimal clinical outcomes in occupation-oriented interventions, emphasizing the importance and mutual contribution of both processes. On the action side, integration of a new intervention into a clinical practice should be conceptualized as an ongoing process. This should involve careful planning of implementation and prolonged monitoring of both implementation and outcomes, while considering the interplay between intervention structure, clinical setting, and clients’ and practitioners’ characteristics. In this way, it becomes possible to reveal the best method by which to adapt the intervention for each setting. Still, the knowledge creation process should be built in an appropriate way; it must be supportive of the clinical actions, through clear formulation of active intervention components for fidelity, systematic evaluation of the extent of contextual factors’ impacts on the intervention’s effectiveness, and definitions of which types of outcomes will be reasonable to obtain, under which conditions and with specific populations. These goals can be achieved by, for example, naturalistic studies. It was shown that, in the case of OC, improvement in subjective participation-related outcomes and experiences of recovery orientation depended on the structure of the implementation process, type of service, intervention dosage, and clients’ factors, as aspects of clinical action. Meanwhile, objective outcomes’ improvements were more resistant to the influence of clinical action aspects. Strategies for closing the gap between what we know from research and what we do in practice will be critical in efforts to provide more effective services for clients, consistent with a professional role and supported in a valuable manner by research and evidence.

## Figures and Tables

**Table 1:** Summary of the descriptive parameters by the studies (N = 82).

	1st Study	2nd Study	3rd Study
Design	Pre-post, comparative	Pre-post, comparative	Cross-over
Participants			
N (study/control)	33 (16/17)	30 (14/16)	19
Health condition	Schizophrenia—84.8% Schizoaffective dis.—15.2%	Schizophrenia—70% Schizoaffective dis.—30%	Affective dis.—47.4%; Anxiety—31.6%; Personality dis.—15.8% Schizophrenia—5.2%
Gender	87.9% women	75.7% men	68.4% women
Age: M(SD)	33.7 (SD = 9)	32.7 (SD = 9.9)	32.3 (SD = 12.5)
Years of illness: M(SD)	10.3 (7.1)	10.3 (7.9)	5.3 (5.5)
Setting	In-patient acute wards	In-patient acute wards	Day-care ward
Group facilitator	OT, completed an OC workshop, under ongoing supervision	OT, completed an OC workshop, with occasional supervision	OT, completed an extensive OC workshop, with occasional supervision
Intervention delivery details	Cyclic group of 10 sessions, rigorous keeping of intervention concepts. Median rate of participation = 4 sessions	Cyclic group of 10 sessions, rigorous keeping of intervention procedures Median rate of participation = 5 sessions	Cyclic group of 8 sessions, choose based on experts’ panel, rigorous keeping of intervention concepts. Participation rate = 8 sessions

**Table 2:** Summary of the results from the effectiveness studies: patterns of change (N = 82) *.

		1st Study: Pre-Post, Comparative		2nd Study: Pre-Post, Comparative		3rd Study: Cross-Over	
		Study	Control	Study	Control	Intervention	Control
Statistics		t-/Nonparametric Tests		t-/Nonparametric Tests		Repeated Measures	
Measures							
Secondary outcomes	Cognition						
	Distinct cognitive constructs	↑↑↑	↑↓	↑↑	↑↑	NA	NA
	General cognitive functioning	NA	NA	NA	NA	-	-
	Symptoms	↑↑↑	↑	↑↑	↑↑	NA	NA
	Functional capacity	↑	-	↑	-	↑	-
	Participation						
	diversity	↑	-	-	-	↑	-
	intensity	-	-	-	-	-	-
	enjoyment	-	-	-	-	↑	-
	satisfaction	-	-	-	-	-	-
Primary outcomes	Recovery orientation (RSA)	↑	-	-	↑	NA	NA
	Intention for participation	↑	-	↓	-	NA	NA
	Experience of Autonomy (IPA)	-	-	↓	↑↑	↑↑↑	-

* Notes: The number of arrows indicates a number of measurements where the change was found; “↑”—statistical significant improvement in pre-post evaluation; “↓”—statistical significant decline in pre-post evaluation; “-”—no statistical significant change in pre-post evaluation; NA—parameters that were not measured in the study; RSA—Recovery Self-Assessment; IPA—the Impact on Participation and Autonomy.

## Data Availability

The data will be available upon the request from the corresponding author.

## Supplementary Materials

The following supporting information can be downloaded at: https://www.mdpi.com/article/10.3390/ijerph20032141/s1, File S1: Description of the measurements [11,12,29,30,31,32,33,34,35,36,37,38,39].
