# Supplementary material for: Closing the Gap: From Research to Practice in Mental Health Interventions
Source: Int J Environ Res Public Health. 2023 Jan 24;20(3):2141. doi: 10.3390/ijerph20032141 (PMC9915162; doi:10.3390/ijerph20032141)
Supplement: Supplementary file 1 [file ijerph-20-02141-s001.zip › ijerph-2101937-supplementary.pdf]

File S1: Description of the measurements.

The primary outcomes were set with a focus on participation and recovery dimensions that were addressed with (I) the Intention to Participate Scale for evaluation of the intention to participate in meaningful activities, (II) the Impact on Participation and Autonomy tool (IPA) assessing experience of autonomy in activities and occupations, and (III) the Recovery Self-Assessment for evaluation of the experience of service as recovery-oriented. The secondary outcomes addressed personal factors and included measurement of (I) dimensions of participation in daily occupations, as were assessed with the Adults Subjective Assessment of Participation, (II) functional capacity in the IADL (was assessed with the Observed Tasks of Daily Living-Revised, (III) aspects of cognitive functioning (were evaluated using the Neurobehavioral State Cognitive Assessment; Trail Making Test, Part A and Part B; Rey-Osterrieth Complex Figure test; and Category Fluency Test) or general cognitive status (was evaluated with Modified Mini-Mental State Examination), and (IV) the severity of the illness' symptoms (the Positive and Negative Syndrome Scale), for first and second studies only. The full description of the tools can be find in the Appendix 1. The detailed description of the evaluation procedures can be found in our previous publications [1,2].

Primary outcome measurement

a. Impact on Participation and Autonomy (IPA) [3] is a 32-item questionnaire to quantify experience of autonomy in participation in 5 activity areas: indoors activities, family roles, outdoors activities, social life and relationships, and work and education. Each item is scored using a 5-point Likert scale, ranging from 0 (very good) to 4 (very poor) with higher scores representing poorer participation and autonomy. The average score for each subscale was calculated.

The IPA is a reliable and validated assessment. Cronbach's alpha for the IPA domains ranged between 0.81 and 0.91, indicating good homogeneity. On item level, weighted Kappa ranged between 0.56 and 0.90. On domain level, the test-retest reliability of the IPAQ was good: intraclass correlation coefficients ranged between 0.83 and 0.91. Convergent validity was largely supported by the correlations between 4 similar domains of the London Handicap Scale (LHS) and the IPA scores (e.g. "social relations" and "autonomy outdoors" IPA subscales and "social integration" LHS subscale). Discriminant validity was best demonstrated by low correlations between the specific IPAQ subscales and domains of the LHS representing theoretically different constructs (e.g. "social relations" (IPAQ) and "economic self-sufficiency") [3].
Rasch rating scale analysis confirmed the internal scale validity of the IPA for use in persons with mental disorders [4].

b. Intention for Participation Scale (IPS) [5] is estimate the level of intention to participate in activities within 10 categories of personal meaning by a visual analog scale (0–10) [6]. The content validity of the scale was established based on reports of 10 occupational therapists and 15 people with serious mental illness with agreement of 80% up to 96% among the items. Internal consistency of the scale was demonstrated to be sufficient [2].

c. Recovery Self-Assessment (RSA) [7]: A 36-item self-reported inventory managed to estimate the degree to which the service is perceived as recovery-oriented in 5 domains: life goals, involvement, diversity of treatment options, choice, and individually tailored services (5-point Likert scale).

Secondary outcome measures

The participants were assessed, as secondary outcomes, for the following parameters: their cognitive functioning was evaluated using (a) Trail Making Test (TMT) Part A and Part B [8] for speed of processing and executive functioning evaluation; (b) Neurobehavioral Cognitive Status Examination (Cognistat) [9] for attention, verbal memory, language understanding, constructional abilities, and reasoning (orientation, attention, understanding, construction, memory, similarities, and judgement sub-tests); and (c) Category Fluency Test (CFT) [10] for speed of processing evaluation. Schizophrenia symptoms (general psychopathology symptoms, positive and negative symptoms) were evaluated using the Positive and Negative Syndrome Scale (PANSS) [11]. The following occupation and participation measures were selected for this study: (a) Observed Tasks of Daily Living-Revised (OTDL-R) [12] for estimation of functional capacity in three Instrumental Activities of Daily Living (IADL) domains based on performance: medication management, financial management, communication and telephone usage; and (b) Adults Subjective Assessment of Participation (ASAP) [13] for evaluation of participation patterns based on 52 activities organized in 10 categories (of everyday life activities: domestic life, entertainment and recreation, taking care of children and other adults, learning and applying knowledge, physical activity and sport, self-care, quite recreation, religion and spirituality, and vocation) with four scores for each category: intensity (number of actual activities done), diversity (frequency of activities), satisfaction with performance and enjoyment.

References

1. Lipskaya-Velikovsky, L.; Kotler, M.; Krupa, T. Description of and Preliminary Findings for Occupational Connections, an Intervention for Inpatient Psychiatry Settings. *Am. J. Occup. Ther.* **2016**, *70*, 14688. https://doi.org/10.5014/ajot.2016.014688.
2. Lipskaya-Velikovsky, L.; Krupa, T.; Silvan-Kosovich, I.; Kotler, M. Occupation-focused intervention for in-patient mental health settings: Pilot study of effectiveness. *J. Psychiatr. Res.* **2020**, *125*, 45–51. https://doi.org/10.1016/j.jpsychires.2020.03.004.
3. Cardol, M.; de Haan, R.J.; de Jong, B.A.; van den Bos, G.A.; de Groot, I.J. Psychometric properties of the Impact on Participation and Autonomy Questionnaire. *Arch. Phys. Med. Rehabil.* **2001**, *82*, 210–216.
4. de Vries-Uiterweerd, A.; Flotho, W. Validity of the Impact on Participation and Autonomy (IPA) Questionnaire for Use in People with Mental Disorders: A Cross-National Study. Thesis from European Master of Science: Occupational Therapy. 2007. Available online: http://www.ot-euromaster.nl/docs/abstracts.pdf (accessed on 10 October 2022).
5. Krupa, T.; Edgelow, M.; Chen, S.; Mieras, C.; Almas, A.; Perry, A.; Radloff-Gabriel, D.; Jackson, J.; Bransfield, M. *Action over Inertia: Addressing the Activity-Health Needs of Iindividuals with Serious Mental Illness*; CAOT Publications ACE: Ottawa, ON, Canada, 2007.
6. Moll, S.E.; Gewurtz, R.E.; Krupa, T.M.; Law, M.C. Promoting an occupational perspective in public health. *Can. J. Occup. Ther.* **2013**, *80*, 111–119.
7. O'Connell, M.J.; Tondora, J.; Kidd, S.A.; Stayner, D.; Hawkins, D.; Davidson, L. RSA-R, Person in Recovery, Family Member/Significant Other, Administrator/Manager, and Provider Versions. 2007. Available online: https://medicine.yale.edu/psychiatry/prch/tools/rec_selfassessment/ (accessed on 10 October 2022).
8. Gaudino, E.A.; Geisler, M.W.; Squires, N.K. Construct validity in the Trail Making Test: What makes Part B harder? *J. Clin. Exp. Neuropsychol.* **1995**, *17*, 529–535. https://doi.org/10.1080/01688639508405143.

9. Mitrushina, M.; Abara, J.; Blumenfeld, A. Aspects of validity and reliability of the Neurobehavioral Cognitive Status Examination (NCSE) in assessment of psychiatric patients. *J. Psychiat. Res.* **1994**, *28*, 85–95. https://doi.org/10.1016/0022-395690037-X.
10. Acevedo, A.; Loewenstein, D.A.; Barker, W.W.; Harwood, D.G.; Luis, C.; Bravo, M.; Hurwitz, D.; Aguero, H.; Greenfield, L.; Duara, R. Category fluency test: Normative data for English- and Spanish-speaking elderly. *J. Int. Neuropsychol. Soc.* **2000**, *6*, 760–769. https://doi.org/10.1017/S1355617700677032.
11. Kay, S.R.; Fiszbein, A.; Opler, L.A. The positive and negative syndrome scale (PANSS) for schizophrenia. *Schizophr. Bull.* **1987**, *13*, 261–276. https://doi.org/10.1093/schbul/13.2.261.
12. Diehl, M.; Marsiske, M.; Horgas, A.L.; Rosenberg, A.; Saczynski, J.S.; Willis, S.L. The Revised Observed Tasks of Daily Living: A performance-based assessment of everyday problem solving in older adults. *J. Appl. Gerontol.* **2005**, *24*, 211–230. https://doi.org/10.1177/0733464804273772.
13. Jarus, T.; Barnea, R.N.; Waserlauf, L.; Burtz, S.; Yakoel, I.; Gal-On, L.; Grinbaum, S. The development of the Israeli Adults assessment of participation. *Isrel. J. Occup. Ther.* **2005**, *15*, 93–111.
